# The Effects of Moxibustion on PD-1/PD-L1-Related Molecular Expression and Inflammatory Cytokine Levels in RA Rats

**DOI:** 10.1155/2021/6658946

**Published:** 2021-12-11

**Authors:** Yumei Zhong, Deli Lai, Linlin Zhang, Wenting Lu, Yanan Shang, Haiyan Zhou

**Affiliations:** ^1^Chengdu First People's Hospital, Chengdu Integrated TCM & Western Medicine Hospital, Chengdu 610095, China; ^2^Acupuncture and Tuina School, Chengdu University of Traditional Chinese Medicine, Chengdu 610075, China; ^3^The Affiliated Hospital, School of Medicine, UESTC, Chengdu Women's and Children's Central Hospital, Chengdu 610014, China

## Abstract

**Objective:**

Rheumatoid arthritis (RA) is an autoimmune disease that starts with inflammation of the synovium. The pain and joint dysfunction caused by RA urgently need an effective treatment to alleviate the inflammatory reaction and delay the progression of the disease. The pathological damage of RA is proposed to associate with the dysfunction of the programmed cell death 1/programmed cell death ligand 1 (PD-1/PD-L1) pathway. Moxibustion, as a main complementary therapy of traditional Chinese medicine (TCM), has been proved effective to reduce chronic inflammatory reaction on RA, but whether the anti-inflammatory effects are mediated by PD-1/PD-L1 pathway is still unclear. Therefore, moxibustion was conducted in the rats with RA to investigate its effect on PD-1/PD-L1.

**Methods:**

The rats' right hind paws were injected with Freundʼs complete adjuvant (FCA) to establish the model of RA. Seven days after the injection of FCA, moxibustion therapy was performed on the acupoints of Shenshu (BL23) and Zusanli (ST36) once a day for three weeks. Then, ELISA and immunohistochemical methods were used to analyze the influence of moxibustion on the expression of PD-1/PD-L1. If the moxibustion had an effect on the expression of PD-1/PD-L1-related molecules, we would knock down PD-1 with adenovirus vector. After moxibustion therapy, ELISA and histological analysis were performed to observe the anti-inflammatory effect of moxibustion.

**Results:**

The results demonstrated that moxibustion had an effect on the expression of PD-1-related molecules. The results of ELISA manifested that moxibustion decreased the level of IFN-*γ* and increased the level of IL-4 and IL-10. HE staining revealed that moxibustion alleviated the proliferation of synovial tissue. However, the anti-inflammatory effect and pathological improvement were weakened when PD-1 was blocked.

**Conclusions:**

The results indicate that moxibustion affected the expression of PD-1/PD-L1-related molecules and can effectively treat RA damage. The anti-inflammatory effect of moxibustion was weakened when PD-1 was knocked down.

## 1. Introduction

Rheumatoid arthritis (RA) is an autoimmune disease mediated by T cells. Joint synovium is the target tissue damaged by RA. Persistent and chronic synovitis is the main symptom in the pathological process of RA. With the progression of the disease, cartilage destruction and bone erosion may occur, eventually leading to severe disability [1]. The incidence of RA varies among countries and areas of the world, ranging from 0.5% to 1% [2]. Although the pathogenesis of RA is still unclear, previous studies have revealed that several factors including gene, infection, environment, and hormone are related to the pathological damage [3]. The RA treatment aims to inhibit inflammation, relieve pain, and ultimately alleviate synovial hyperplasia and cartilage destruction.

The pathology of RA begins with synovitis that is closely related to the disease progression and functional outcome of RA. The excessive activation of T cell is crucial in the pathological process of RA [4]. The activation and immune response of T cells, such as T helper 1 cell (Th 1) and T helper 2 cell (Th 2), are essential for the emergence and development of RA synovitis [5]. However, the mechanism of T cells activation still needs to be clarified. PD-1 is expressed on activated B cells, T cells, and monocytes and acts as a crucial negative regulator of T-cell activation [6]. PD-L1, the ligand of PD-1, inhibits the proliferation and activation of T cells by interacting with PD-1 [7]. Like other costimulatory molecules, PD-1 and PD-L1 not only express on the cell membrane surface called membrane PD-1 (mPD-1) and membrane PD-L1 (mPD-L1) but also exist in soluble forms, which are known as soluble PD-1 (sPD-1) and soluble PD-L1 (sPD-L1), respectively. Previous study has shown that various malignant tumors express PD-L1 and escape from the host immunity by suppressing T-cell activity [8]. Additionally, researches have observed an augmented expression of PD-1 in synovial T cells and macrophages in patients with RA [4]. Fusion protein of activating PD-1 elevates PD-1 activity and inhibits T-cell proliferation and thus alleviates arthritis in arthritis mouse models. The deficiency of PD-1 or PD-L1 exacerbates the symptoms of RA [9]. Thus, the PD-1/PD-L1 pathway plays an important role in the pathogenesis of RA and may become a promising therapeutic target.

Among the RA therapeutic strategies, drug treatment is the most important one. But both anti-inflammatory and biological agents have some drawbacks and may develop drug resistance [10, 11]. However, the pathophysiology of RA is complicated, and the therapeutic effect remains unsatisfactory. As a complementary therapy, moxibustion plays an important role in anti-inflammatory and pain relief, especially in musculoskeletal diseases, such as knee osteoarthritis [12] and cervical spondylosis [13]. Previous researches have shown that moxibustion can increase the number of regulatory T cells in spleen and restore the balance between Th1 and Th2, thus producing anti-inflammatory effect [14, 15]. At the same time, moxibustion can influence the expression of PD-1/PD-L1 and inhibit the activation of T cells [16]. In order to explore whether the moxibustion can affect the expression of PD-1 related-molecule and the effect of moxibustion is related to the PD-1/PD-L1 pathway, we conducted this study.

## 2. Materials and Methods

### 2.1. Animals

Sprague Dawley rats (weight, 200 ± 20 g) including both males and females were provided by Chengdu University of TCM (Chengdu, China). The rats were fed in the room with a temperature of 22–24°C, a humidity of 20%, and natural light-dark cycles. Animals were free to eat and drink. The experiment was approved by the Ethics Committee of Chengdu University of Traditional Chinese Medicine (Permit Number: SCXK 2013–24).

### 2.2. Establishment of RA Rat Model

In the first experiment, animals were divided into three groups (10 rats in each group) randomly including the control group, the RA group, and the group of RA with moxibustion (Mox). In the second experiment, rats were also divided into three groups: control group, PD-1 interference group (PD-1), and PD-1+RA group. In the third experiment, there were five groups: control group, RA group, RA with moxibustion group (Mox), PD-1 interference with moxibustion group (PD-1+Mox), and PD-1+RA group. The RA rat model was established based on previous studies [16, 17]. On the first day of the experiment, the right hind paw of rat was injected with FCA (Sigma-Aldrich) to establish the model of RA. All the rats, except those in the control group and the PD-1 group, were injected with FCA (0.5 ml/kg) [18]. Seven days later, all the rat's feet developed symptoms of inflammation such as redness, swelling, and restricted ability to move. Rats in the control group and PD-1 group received the same volume of saline.

### 2.3. Establishment of RNAi Model of Vector-Mediated Adenovirus Blocks the Expression of PD-1

On the second day of the experiment, the adenovirus-mediated shRNA-rPD-1 (pDC316-EGFP-ShRNA-rPD-1, Wuhan Weinuosai Biotechnology Co., LTD, China) was injected into the right hind paw of rats in the PD-1 group, PD-1+RA group, and PD-1+Mox group with a dose of 10 *μ*l/rat [19]. The rats in the control group were injected with the same dose of saline.

### 2.4. Moxibustion Method

**Figure 1:**
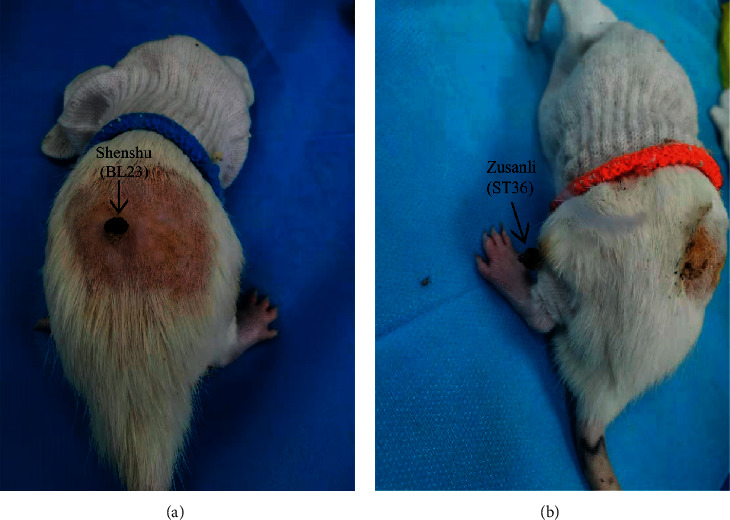
(a, b) Rats receive moxibustion treatment at the acupoints of Shenshu (BL23) and Zusanli (ST36), respectively.

Moxibustion therapy was employed on the eighth day of the experiment. The location of BL23 and ST36 is according to the standards of the Experimental Acupuncture [20]. BL23 is located 7 mm laterally below the spinous process of the second lumbar spine. ST36 is below the knee joint and 6 mm beside the anterior tibial muscle (Figures 1(a) and 1(b)). The rat's fur near the acupoints of BL23 and ST36 was shaved to facilitate moxibustion. Lighting moxa granules Ω (diameter: 2 mm, length: 5 mm) were located on acupoints. Five moxa granules were burned at each point per day by alternating bilateral acupoints for consecutive 3 weeks. There was a day off on the seventh day every week. The rats in the control group, RA group, and PD-1+RA group were fixed for 30 minutes without any therapies.

After treatment for 3 weeks, all rats were anesthetized with isofurane and then sacrificed. 4 ml of blood was extracted from the abdominal aorta and put into a centrifuge tube labeling with serial numbers. After one hour, the supernatant was centrifuged and put into an EP tube stored in −20°C refrigerator. Synovial tissue samples were stored in 4% formaldehyde solution.

### 2.5. Immunohistochemical Examination

Sections were dewaxed and submerged in 3% hydrogen peroxide for ten minutes. Next, the samples were blocked with goat serum for 30 min at room temperature and incubated with PD-1 mouse monoclonal antibody (1 : 50, Mybiosource, California, USA) in blocking solution at 4°C overnight. Later, sections were washed with PBS for three times and incubated with biotinylated goat anti-rabbit IgG (1 : 100, Wuhan BoShide. Bioengineering Co., Ltd., China) secondary antibody at 37°C for 30 min, followed by signal amplification with streptavidin biotin-horseradish peroxidase (37°C, 30 min) and staining with diaminobenzidine (DAB). At last, sections were restained with hematoxylin. The procedure of detecting PD-L1 was the same as that of detecting PD-1. The first antibody used was PD-L1 rabbit anti-rat IgG (1 : 50, Mybiosource, California, USA). Images were captured by using trinocular microscope (Motic, Xiamen, China).

### 2.6. Enzyme-Linked Immunosorbent Assay (ELISA)

The expression of IL-4, IL-10, and IFN-*γ* in serum was tested by ELISA. Next, the researchers took the supernatant and made it in reserve. Then, operations such as the preparation of the standard, sample addition, antibody incubation were conducted according to the corresponding ELISA kit instructions (eBioscience, California, USA), and stop solution was added. Finally, the absorbance value was measured with a microplate analyzer at 450 nm wavelength, and the levels of IL-4, IL-10, and IFN-*γ* in serum of each group were calculated by standard curve. Additional ELISA was also applied to detect the expression of sPD-1 and sPD-L1 in serum samples (ELISA kits: Mybiosource, California, USA).

### 2.7. Histological Analysis

The synovial tissue was fixed in the 4% paraformaldehyde solution. The embedded synovial tissue was sectioned by a microtome to 5 microns and stained with hematoxylin-eosin (HE). HE staining was used to evaluate the proliferation of synovial tissue and the infiltration of inflammatory cell (Tables 1 and 2) [15]. Images were obtained by using trinocular microscope (Motic).

### 2.8. Real-Time PCR Analysis

The mRNA expression of PD-1 was determined by qPCR analysis. Total RNA was extracted from monocytes by using a TRIzol reagent and then reverse-transcribed with a cDNA synthesis kit (Thermo Fisher Scientific) according to the manufacturer's protocol. mRNA expression of *β*-actin and the selected molecules was determined by using SYBR Green Master Mix (Applied Biosystems). Primer sequence was synthesized by Invitrogen (Sangon Biotech, China) as follows: *β*-actin, sense 5ʼ-CGAGTACAACCTTCTTGCTTTCTTGCAGC-3ʼ, and antisense 5ʼ-ACCCATACCCACCATCACAC-3ʼ; PD-1, sense 5ʼ-TGGCGTCTGTGGGTTCTGTG-3ʼ and antisense 5ʼ-CCCCCAGCCTAAGAATTTCC-3ʼ. The PCR cycling reaction conditions were as follows: preincubation at 95°C for 2 min, followed by 40 cycles of denaturation at 95°C for 30 s and 60°C for 30 s, annealing at 55°C for 30 s, and extension at 72°C for 30 s. The relative expression of PD-1 was analyzed with the 2^**−**ΔΔCT^ method.

### 2.9. Statistical Analysis

Data analysis was performed by using SPSS 22.0 software. The data need to pass the normality test and the homogeneity test of variance. If the data were normally distributed and had homogeneous variances, difference between groups would be assessed by one-way ANOVA test followed by LSD post hoc comparison. Kruskal–Wallis test followed by post hoc Mann–Whitney *U* test would be applied when there was lack of homogeneity of variances. Data were presented as the mean ± SD. The *P* value less than 0.05 was thought to be statistically significant.

## 3. Results

### 3.1. The Effect of Moxibustion on sPD-1 and sPD-L1

**Figure 2:**
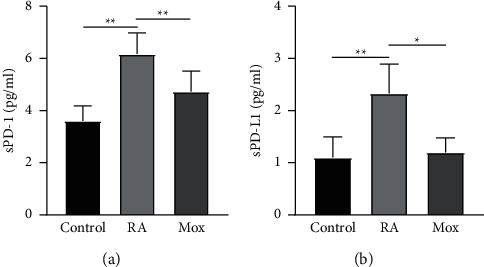
The levels of soluble programmed cell death 1/soluble programmed cell death ligand 1 (sPD-1/sPD-L1). (a, b) The levels of sPD-1 and sPD-L1 in serum, respectively. *n* = 10 for each group. Data were expressed as the mean ± SD.  ^*∗∗*^*P* < 0.01; ^*∗*^*P* < 0.05.

The experiment used ELISA method to observe the levels of sPD-1 and sPD-L1 in serum. Compared with the control group, the levels of sPD-1 and sPD-L1 were significantly increased in the RA group (RA group: sPD-1: 6.17 ± 0.81, sPD-L1: 2.33 ± 0.56 Figures 2(a) and 2(b)). After moxibustion treatment, the level of sPD-1 and sPD-L1 decreased in the Mox group compared with the RA group (Figures 2(a) and 2(b), Mox group: sPD-1: 4.73 ± 0.78, sPD-L1: 1.20 ± 0.28).

### 3.2. The Effect of Moxibustion on mPD-1 and mPD-L1

**Figure 3:**
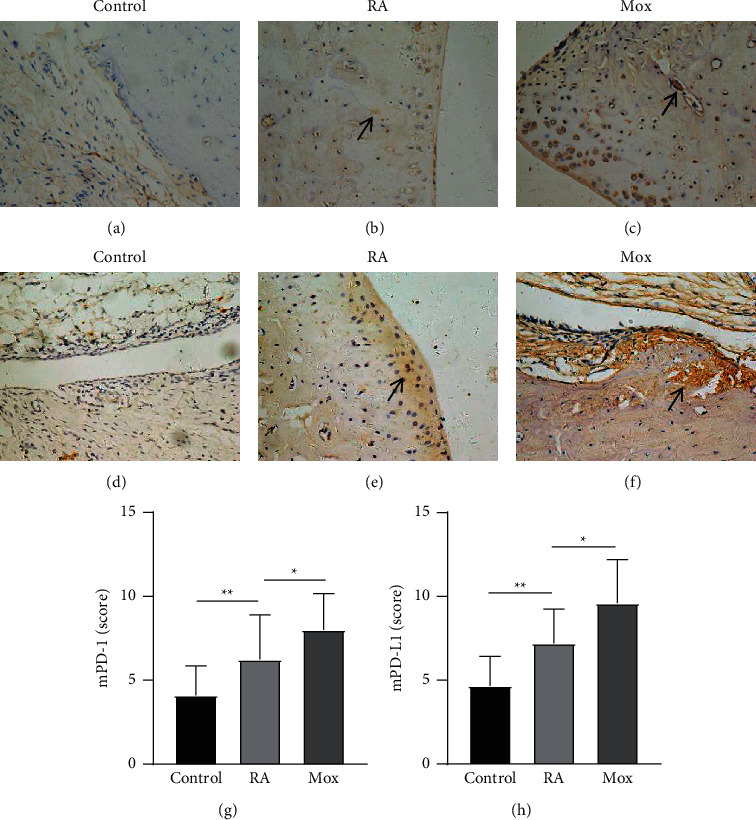
The expression of membrane programmed cell death 1/membrane programmed cell death ligand 1 (mPD-1/mPD-L1). (a–c) The expression of mPD-1 in control (a), RA (b), and Mox groups (c). (d–f) The expression of mPD-L1 in control (d), RA (e), and Mox groups (f). Notes: arrows represent the positive expression of mPD-1 and mPD-L1. Magnification, ×200. (g, h) The score of positive expression of mPD-1 and mPD-L1 in synovial tissue. *n* = 10 for each group. Data were expressed as the mean ± SD.  ^*∗∗*^*P* < 0.01; ^*∗*^*P* < 0.05.

Next, the researcher used immunohistochemical method to observe the expression of mPD-1 and mPD-L1 in synovial tissue. The results showed an increase in the expression of mPD-1 (6.24 ± 2.66) (Figures 3(b) and 3(g)) and mPD-L1 (7.20 ± 2.04) (Figures 3(e) and 3(h)) in the RA group compared with the control group. However, after moxibustion treatment, the expression of mPD-1 (8.00 ± 2.17) (Figures 3(c) and 3(g)) and mPD-L1 (9.60 ± 2.59) (Figures 3(f) and 3(h)) upregulated in the Mox group compared with the RA group.

### 3.3. Vector-Mediated Adenovirus Blocks the Expression of PD-1

**Figure 4:**
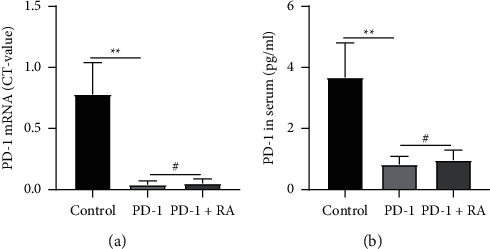
The expression of programmed cell death 1 (PD-1) on adenovirus-mediated PD-1 RNAi. (a) The expression of PD-1 mRNA. (b) The level of PD-1 in serum. *n* = 10 for each group. Data were expressed as the mean ± SD.  ^*∗∗*^*P* < 0.01; ^#^*P* > 0.05.

To investigate the role of PD-1 in the anti-inflammatory effect of moxibustion, PD-1 was blocked by adenovirus in vivo to establish a RNAi model. The efficacy of adenovirus-mediated PD-1 RNAi model was evaluated by qPCR and ELISA techniques. The results suggested that the expression of PD-1mRNA and PD-1 protein in serum was significantly lower in PD-1 and PD-1+RA group (Figure 4(a): PD-1mRNA: 0.04 ± 0.02, 0.051 ± 0.021; Figure 4(b): PD-1 protein: 0.82 ± 0.27, 0.97 ± 0.33) compared with the control group (Figure 4(a): PD-1mRNA: 0.78 ± 0.26; Figure 4(b): PD-1 protein: 3.67 ± 1.13). The results indicated that adenovirus-mediated PD-1 RNAi model was successful.

### 3.4. The Effect of Moxibustion on Pathological Changes of Synovium

**Figure 5:**
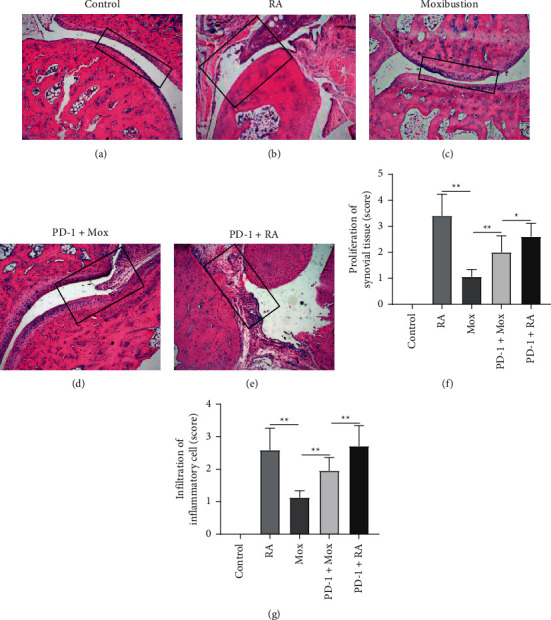
Pathological changes and scores of the joint. HE staining showed the pathological changes of synovial tissue in the figures of control (a), RA (b), Mox (c), PD-1+Mox (d), and PD-1+RA (e) group. Magnification, ×100. (f, g) The score of the proliferation of synovial tissue and infiltration of inflammatory cell. *n* = 10 for each group. Data were expressed as the mean ± SD.  ^*∗∗*^*P* < 0.01; ^*∗*^*P* < 0.05. The square represents the pathological changes of the joint.

Persistent and progressive synovitis exists in the whole pathological process of RA and is an important pathological change. Next, to observe the protective effects of moxibustion on RA, HE staining was performed to assess the pathological morphology of joint. Smooth synovial tissue was observed in the control group (Figure 5(a)). In RA group and PD-1+RA group, obvious proliferation of synovial tissue and infiltration of inflammatory cell were shown in the synovial tissue (Figures 5(b), 5(e), 5(f), and 5(g)). Compared with the RA group, the levels of proliferation and infiltration reduced in the Mox group (Figures 5(c), 5(f), and 5(g)). The pathological morphology of synovial tissue in the PD-1+Mox group was also improved compared with the RA group, but not as obvious as the Mox group (Figures 5(d), 5(f), and 5(g)). The results showed that moxibustion could inhibit the pathological changes of synovitis in rats with RA, but the effect was weakened when PD-1 was interfered.

### 3.5. The Anti-Inflammatory Effect of Moxibustion

**Figure 6:**
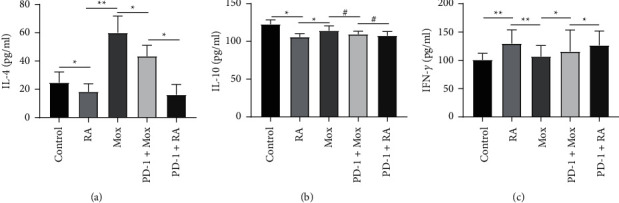
The levels of interleukin-4 (IL-4), interleukin-10 (IL-10), and interferon-*γ* (IFN-*γ*). (a–c) The expression of IL-4, IL-10, and IFN-*γ* in different groups. *n* = 10 for each group. Data were expressed as the mean ± SD.  ^*∗∗*^*P* < 0.01; ^*∗*^*P* < 0.05; ^#^*P* > 0.05.

T cells take part in the inflammatory process of RA mainly by secreting cytokines, such as IFN-***γ*** and IL-4, which is an important link in the development of synovitis [21]. Proinflammatory cytokines can cause proliferation of synovial cells, upregulate the secretory function of synovial cells, and thus aggravate the symptoms of synovitis [22]. The imbalance between Th1 and Th2 cells is closely related to RA. Therefore, we conducted ELISA to analyze the levels of cytokines in serum. Compared with the control group, the levels of IL-4 (18.31 ± 5.57) (Figure 6(a)) and IL-10 (105.78 ± 4.37) (Figure 6(b)) were decreased in the RA group, while the level of IFN-*γ* (129.68 ± 24.13) (Figure 6(c)) was increased. After the treatment with moxibustion, the expression of cytokines changed; in other words, compared with RA group, the expression of IL-4 (60.0 ± 12) and IL-10 (114.23 ± 6.45) was increased, and the expression of IFN-*γ* (106.72 ± 19.34) was decreased. The results of ELISA showed that the level of IL-4 (43.52 ± 7.63) decreased in the PD-1+Mox group compared with the Mox group, and the expression of IFN-*γ* (115.57 ± 27.80) increased, but there was no statistical difference in the level of IL-10 (109.56 ± 3.98). The results indicated that moxibustion could promote the expression of IL-4 and IL-10 and inhibit the expression of IFN-*γ*. In the case of PD-1 knocked down, the effect of moxibustion on the upregulation of the expression of IL-4 and the downregulation of the expression of IFN-*γ* was inhibited, but it had no significant effect on the expression of IL-10.

## 4. Discussion

The abnormal activation of T cells is an important link in the occurrence of RA. In its initial stage of the disease, joint pain and swelling are the main symptoms. As it develops, joint stiffness and deformity may occur [23]. RA is characterized by persistent synovitis, proliferation of synovial tissue, and pannus formation, resulting in the decline of joint function, a worse life quality, and higher direct or indirect medical expenses [24]. Because the cause of RA is still unclear, the efficacy of the current treatment is unsatisfactory. Its treatment includes drug therapy, biotherapy, and physical therapy [25]. Disease-modifying antirheumatic drugs (DMARDs) are one of the most common choices in clinic to prevent the progression of RA, but the drug has a slow onset and takes a long time [26]. Nonsteroidal anti-inflammatory drugs (NSAIDs) and glucocorticoids (GC) have good anti-inflammatory effects in the active phase of RA and could relive joint pain at the same time. Biologics could effectively alleviate joint swelling and stiffness of RA patients and inhibit the secretion of proinflammatory factors [27]. However, the high cost of it will undoubtedly increase the economic burden of patients. Taking DMARDs and NSAIDs for a long term may cause gastrointestinal reactions and increase the risk of cardiovascular diseases [26]. As a result, RA patients on routine medication are turning to other substitutes, such as the complementary and alternative medicine [28].

Moxibustion is an external therapy based on the theory of TCM. The researchers conducted a large number of comparative studies on the morphology of acupoints from the view of anatomy and found that animal acupoints have similar structures to human being such as nerves, vessels, lymph, and mast cells [29]. The researchers observed the gastric peristalsis in animals by electro-acupuncture at ST36 and nonmeridian points. The results showed that the ST36 can work [30]. Meanwhile, acupuncture at BL23 can change the pressure in the bladder, but the nonmeridian points cannot. Numerous studies have shown that animal acupoints are similar to human acupoints in terms of positioning and function, and they are good carriers for scientific research [31]. However, due to the differences in species and evolutionary process, they cannot be completely regarded as human acupoints. As a supplementary therapy, its effective mechanism may be associated with the thermal effects, radiation effects, and pharmacological actions produced by the burning of moxa and the influence of meridians and acupoints. The theory of TCM holds that moxibustion can invigorate qi and stimulate blood circulation, warm meridians, and regulate yin and yang [32]. Previous results suggested that moxibustion thermal stimulation affected circulation of the blood and regulated the function of the nerves [33]. In present study, researchers observed the effect of moxibustion on PD-1/PD-L1-related molecules and preliminarily studied the mechanism of moxibustion in preventing inflammation.

Overactivated T cells are crucial in the pathological process of RA. The combination of PD-1 by PD-L1 can inhibit the T-cell receptor-mediated lymphocyte proliferation and cytokine secretion [34]. The activation of PD-1/PD-L1 pathway can largely reduce the damage of tissues and organs caused by autoimmune reaction [35]. However, the effect of the PD-1/PD-L1 pathway is thought to be bidirectional [36]. Previous study found that the elevated plasma levels of sPD-1 in early RA and the inverse correlation with total Sharp score (TSS) suggest that sPD-1 is an important mediator in inflammatory disease progression [37]. In patients with chronic RA, the plasma level of sPD-1 was also significantly higher than in healthy volunteers [37]. Studies observed that the expression of sPD-1/sPD-L1 increased in serum of RA patients, and the expression of mPD-1/mPD-L1 also increased in synovial tissue and spleen [38, 39]. CD4+T cells were cocultured with synovial fluid mononuclear cells in vitro, and the addition of high concentration of sPD-L1 fusion protein could promote the proliferation of T cells [4]. In this study, an obvious decrease in sPD-1 and sPD-L1 and an increase in mPD-1 and mPD-L1 in the Mox group were observed. sPD-1 is reported to exhibit functional antagonism, thereby inhibiting the immune regulatory effects of the membrane-bound PD-1 and PD-L1 and continuing the activation of the T cells [4, 36]. The research results of Wan et al. showed that the inhibition of signaling pathway function was due to the competitive binding effect of sPD-1 and sPD-L1 [4]. This study also confirmed that sPD-1 and sPD-L1 could block the inhibition effect of PD-1/PD-L1 signal, thus promoting the abnormal activation and proliferation of T cells. In RA pathology, due to continuous antigen stimulation, the body reacts to inhibit the proliferating of T cells, which promotes the enhancement of negative costimulatory signals, and the expressions of mPD-1 and mPD-L1 are also increased correspondingly. Studies have found that moxibustion can upregulate the expression of anti-inflammatory cytokines (such as IL-4 and IL-10) and downregulate the level of proinflammatory cytokines (such as TNF-*α* and IL-1*β*) at the same time, thus restoring the balance of Th1/Th2 [40, 41]. As a key negative costimulatory molecule, PD-1 plays an important role in the activation of T cells [42]. Moxibustion can inhibit the secretion of sPD-1 and sPD-L1, thus reducing the blocking effect on the PD-1/PD-L1 signaling pathway. Meanwhile, the expression of mPD-1 and mPD-L1 was increased, and the PD-1/PD-L1 signaling pathway was strengthened to exert its immune regulatory function and anti-inflammatory effect. This is what we observed in the early stages of the experiment, and the specific mechanism will be developed with further researches. Based on the effect of moxibustion on the expression of PD-1/PD-L1-related molecules, next, we blocked the expression of PD-1 in RA rat model with adenovirus vector to preliminarily explore whether the effect of moxibustion is related to PD-1.

Synovium is the target tissue damaged by RA. The main pathological features are the proliferation of synovial cells and the infiltration of inflammatory cells dominated by T cells [43]. Synovial tissues are always in a state of inflammation, which may be an important link in the immune-mediated destruction of bone and cartilage [44]. Observation of the pathological morphology and pathological degree of synovial tissue in RA rats by HE can offer a clear understanding about the pathological degree of RA synovial tissue and the effect of moxibustion on synovitis. In this study, researchers found that the moxibustion alleviated the pathological progression of RA. The degree of synovial tissue hyperplasia in the Mox group and PD-1+Mox group was reduced compared with the RA group and PD-1+RA group, but the effect of the PD-1+Mox group is not as good as that of the Mox group. Previous studies have revealed that moxibustion can alleviate the knee swelling and repair the eroded cartilage in RA animals [14, 15, 45]. Based on that, our results suggest that moxibustion could inhibit the pathological changes of synovitis, but the inhibitory effect is decreased when PD-1 is blocked.

Th1/Th2 cytokines in normal people maintain a state of dynamic balance. In RA patients and animal models, the balance of T cell subpopulation differentiation was broken, resulting in the imbalance of Th1/Th2 cytokines [46]. The imbalance of cytokines was characterized by secreting more proinflammatory cytokines and less anti-inflammatory cytokines. It was found that the level of serum IFN-*γ* was increased and the level of IL-4 was decreased in patients with RA in active stage and remission stage [44]. Previous studies demonstrated that moxibustion at ST36 and BL23 could decrease the level of TNF-*α* and alleviate the destruction of bone and cartilage in RA model [17, 45]. In this study, the expression of IFN-*γ* in the Mox group decreased, and the level of IL-10 and IL-4 was higher compared with the RA group. However, the abovementioned effect was not distinctly obvious in the PD-1+Mox group when compared with the Mox group. In this study, moxibustion treatment can reduce the content of IFN-*γ* and increase the content of IL-10 and IL-4 and effectively regulate and restore the imbalance of Th1 and Th2. But, in the case of PD-1 interference, the above effects were weakened. It suggested that the anti-inflammatory effect of moxibustion on RA and the effect of restoring Th1/Th2 cytokines balance may be related to the negative regulation of PD-1 on T cells activation. In conclusion, moxibustion had a good anti-inflammatory effect on the RA rat. However, the specific mechanism of moxibustion in treating RA remains further researches to clarify.

## 5. Conclusion

The present study showed that moxibustion could affect the expression of PD-1/PD-L1-related molecules and had a good anti-inflammatory effect on the rat model of RA. However, in the case of PD-1 interference, the anti-inflammatory effect was weakened.

## Figures and Tables

**Table 1:** Presentation of synovial tissue proliferation.

Staging	Presentation of synovial tissue proliferation	Score
Normal	Synovial tissue is smooth; synovial lining layer cells are flat monolayer, or they are shown with 2-3 layers and are arranged in regular order.	0 (−)
Mild	Edema appears in the synovial interstitium; vascular dilatation emerges; endothelial cells are swollen; synovial macrophages can also be seen.	1∼2 (+∼++)
Moderate	Synovium is obviously thickened and can be up to 10 layers; vascular hyperplasia appears; the tube wall is thickened; and thrombosis develops.	3 (+++)
Severe	Synovial papillae appear; pannus is formed at cartilage junction; fibrous granuloma or scar is formed; cartilage and bone are destructed.	4 (++++)

**Table 2:** Presentation of inflammatory cell infiltration.

Staging	Presentation of inflammatory cell infiltration	Score
Normal	Inflammatory cells are detected occasionally	0 (−)
Mild	Inflammatory cells are scattered or form lymphatic follicles	1∼2 (+∼++)
Moderate to severe	Inflammatory cells gather together; numerous fibroblasts and lymphocytes can be seen; macrophages cluster together	3∼4 (+++∼++++)

## Data Availability

The data supporting the results of this study are available from the corresponding authors. The email address is zhouhaiyan@cdutcm.edu.cn.

## Additional Points

The research just observed that moxibustion had an effect on the expression of PD-1/PD-L1-related molecules. However, the reason why moxibustion has different effects on PD-1-related molecules has been ignored.
